# Knowledge Gaps Among Brazilian Healthcare Professionals Regarding Oropouche Virus: A National Cross-Sectional Study

**DOI:** 10.3390/healthcare13172192

**Published:** 2025-09-02

**Authors:** Layna de Cássia Campos Cravo, Raimunda do Socorro da Silva Azevedo, Lívia Medeiros Neves Casseb, Jannifer Oliveira Chiang

**Affiliations:** Department of Arbovirology and Hemorrhagic Fevers, Evandro Chagas Institute, Rodovia Br 316, Km 07, S/N, Ananindeua 67030-000, Pará, Brazil; layna.campos@hotmail.com (L.d.C.C.C.); raimundaazevedo@iec.gov.br (R.d.S.d.S.A.); liviacasseb@iec.gov.br (L.M.N.C.)

**Keywords:** Oropouche virus, *Orthobunyavirus oropoucheense*, healthcare professionals, Brazil, public health

## Abstract

Background/Objective: Oropouche virus (OROV—*Orthobunyvirus oropoucheense*) is a neglected arbovirus endemic to parts of Latin America, particularly the Brazilian Amazon. Despite its increasing epidemiological relevance, little is known about healthcare professionals’ awareness and preparedness regarding the virus. Methods: A cross-sectional, questionnaire-based study was conducted between February 2024 and March 2025 with 760 healthcare professionals across 21 Brazilian states. Participants represented various professional categories, including nursing, medicine, pharmacy, and biomedical sciences. The survey assessed knowledge on OROV epidemiology, clinical recognition, diagnostic practices, and compulsory notification. Results: Among participants, 37.4% had never heard of OROV, and 38.0% had heard of it but knew little about it. Most professionals first learned about the virus within the past year (31.8%). The majority (86.1%) reported not having received information about OROV during undergraduate education. Only 21.9% knew which diagnostic test to request, and 64.7% were aware that OROV is subject to mandatory notification. Notably, 71.2% were unaware of the virus’s potential neurological complications. Conclusions: These findings reveal a significant gap in the education and training of healthcare professionals regarding OROV, even in endemic areas. The results underscore the need for curricular reform, expanded continuing education, and stronger integration of OROV into national epidemiological surveillance efforts.

## 1. Introduction

Oropouche virus (OROV—*Orthobunyavirus oropoucheense*) is an emerging arbovirus endemic to parts of Central and South America, particularly in the Amazon Basin. Belonging to the *Peribunyaviridae* family, *Orthobunyavirus* genera. OROV is transmitted primarily by *Culicoides paraensis* midges and can cause outbreaks of Oropouche fever, a dengue-like illness characterized by acute febrile symptoms and, in some cases, neurological complications such as meningitis and encephalitis. The virus maintains a sylvatic transmission cycle involving wild animals, especially sloths, monkeys, and rodents, and hematophagous arthropods, and can spill over into urban cycles when competent vectors are present. Since its first identification in Trinidad in 1955 and later detection in Brazil in 1961, OROV has caused over half a million reported cases, although its true burden is likely underestimated due to underreporting and misdiagnosis [1,2,3].

For decades, Oropouche fever remained a neglected topic in Brazil’s public health agenda, overshadowed by other arboviral diseases such as dengue, Zika, and chikungunya. However, the situation changed in 2024 when Brazil experienced an alarming surge in OROV cases, including the first officially reported deaths and reports of neurological sequelae, spontaneous abortions, and possible associations with congenital anomalies. These developments prompted renewed scientific interest and raised concerns about the preparedness of healthcare systems to respond effectively to the virus [4].

The expanding geographic range of OROV is facilitated by urbanization, deforestation, and climate change, all of which increase human exposure to the vector. *Culicoides paraensis*, one of the main vectors of OROV in urban areas, typically breeds in moist, shaded environments with abundant organic matter, such as wet soil, tree holes, decomposing vegetation, and poorly drained urban waste. Deforestation alters natural habitats, pushing vector populations closer to human dwellings, while urbanization leads to the accumulation of breeding sites near residential areas. Additionally, climate change may expand the geographical and seasonal distribution of *C. paraensis*, increasing the risk of outbreaks in new regions [5,6]. In this context, early recognition of symptoms, timely diagnosis, and mandatory reporting are essential to prevent further spread and manage outbreaks. Nonetheless, there is limited data on the level of awareness and preparedness of Brazilian healthcare professionals regarding OROV detection, clinical management, and public health response, particularly in endemic regions where the virus poses a growing threat.

The northern region of Brazil, particularly the Amazon Basin, is considered the epicenter of OROV activity. Historically, this region has reported the highest number of OROV outbreaks, with confirmed epidemics in states such as Pará, Amazonas, Acre, and Rondônia. Belém, the capital of Pará, experienced significant outbreaks in the 1980s and continues to report OROV circulation. The virus was first isolated in Brazil in 1961, and more than 30 outbreaks have been documented since then. It is estimated that over 500,000 people have been affected by Oropouche fever in Brazil between 1961 and 2019, primarily in the Amazon region. However, underreporting is common due to limited routine testing and clinical overlap with other arboviruses such as dengue, Zika, and chikungunya. Recent surveillance data indicate a resurgence of OROV, with outbreaks reported between 2022 and 2024 in both rural and urban municipalities across the Amazon. For example, in Pará alone, over 3800 suspected cases were recorded during this period, underscoring the virus’s continued public health relevance and its potential for urban expansion [7].

This study aimed to evaluate the knowledge of healthcare professionals in Brazil regarding OROV, including epidemiological, clinical, and diagnostic aspects. By identifying knowledge gaps, the findings can inform strategies to improve surveillance, education, and the clinical management of this re-emerging arboviral threat.

## 2. Materials and Methods

This was a descriptive, cross-sectional study conducted between February 2024 and March 2025 in Brazil. The target population included healthcare professionals from various fields, including nursing, medicine, pharmacy, biomedical sciences, physiotherapy, nutrition, biology, and health management. Participation was voluntary, and all respondents provided informed consent prior to data collection.

A purposive sampling strategy was employed, initially stratified by professional category, with a primary focus on nursing and medicine due to their central roles in clinical care. As the study progressed, other health-related professions were incorporated to capture a broader perspective on awareness and preparedness. Inclusion criteria were (i) being a health professional and (ii) being actively involved in clinical practice or public health settings. Individuals who were not working in the health sector or who did not provide digital consent were excluded.

Data were collected through an online questionnaire disseminated via institutional emails, professional councils, academic networks, and social media platforms. The survey instrument included both closed and semi-structured questions addressing sociodemographic characteristics (sex, professional category, region of work), academic and professional background (years since graduation, specialization), and specific knowledge about OROV, including awareness of the virus and its clinical features, time since first exposure to information about OROV, understanding of notification requirements, familiarity with diagnostic testing, and awareness of neurological complications.

The questionnaire was pretested by a panel of experts to ensure clarity, relevance, and content validity.

Data were analyzed using descriptive statistics, including absolute and relative frequencies. Cross-tabulations were performed to explore associations between professional characteristics and knowledge variables. The analysis was conducted using Microsoft Excel (version 16.0. Redmond, WA, USA, Microsoft Corporation; 2018) and GraphPad Prism (version 9.0. San Diego, CA, USA, GraphPad Software, Inc.; 2020). Figures and tables were generated to illustrate key findings.

## 3. Results

### 3.1. Participant Characteristics

**Figure 1:**
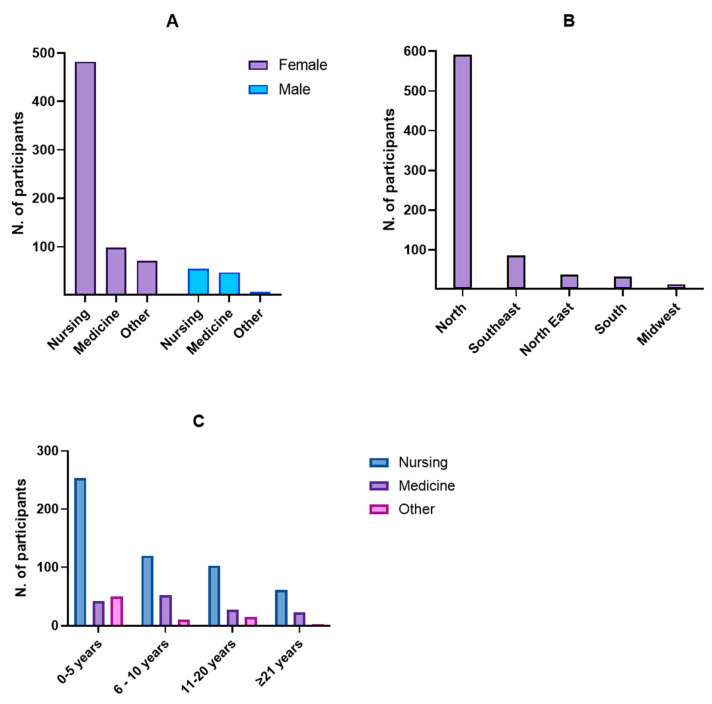
Sociodemographic and professional characteristics of the study participants. (**A**) Distribution of participants by professional category and sex. (**B**) Geographic distribution of participants by Brazilian region. (**C**) Distribution of participants by years since graduation.

A total of 760 healthcare professionals participated in the study. The sample consisted primarily of nursing (*n* = 537; 70.7%), followed by medicine professionals (*n* = 145; 19.0%) and other health-related categories such as pharmacy, biomedicine, and nutrition (*n* = 78; 10.3%). Most participants were women (*n* = 651; 85.6%), with female nurses comprising the largest subgroup (Figure 1A).

Participants were geographically distributed across 21 of Brazil’s 27 federative units. The majority were from the Northern region (*n* = 592; 77.9%), followed by the Southeast (*n* = 86; 11.3%), Northeast (*n* = 37; 4.9%), South (*n* = 32; 4.2%), and Midwest (*n* = 13; 1.7%) (Figure 1B). The state of Pará accounted for the largest number of respondents (*n* = 554; 72.9%), reflecting the virus’s endemic nature in the Amazon region. Table 1 presents the absolute and relative frequencies of participants by state and region. When analyzing knowledge about OROV by state, we found that the highest proportions of professionals unaware of the virus were located in states from the South and Southeast regions, such as Paraná (72.2%), Rio Grande do Sul (68.4%), and São Paulo (61.9%). In contrast, professionals from northern states, including Amazonas and Pará, demonstrated higher awareness levels, with only 25.8% and 33.1% reporting no prior knowledge of OROV, respectively.

In terms of professional experience, 345 participants (45.4%) had graduated within the past five years, 183 (24.1%) between six and ten years ago, 145 (19.1%) between eleven and twenty years ago, and 87 (11.4%) more than twenty-one years ago (Figure 1C). Additionally, 516 professionals (67.8%) reported having a postgraduate specialization, while 244 (32.2%) did not.

### 3.2. Knowledge and Awareness of Oropouche Virus

**Figure 2:**
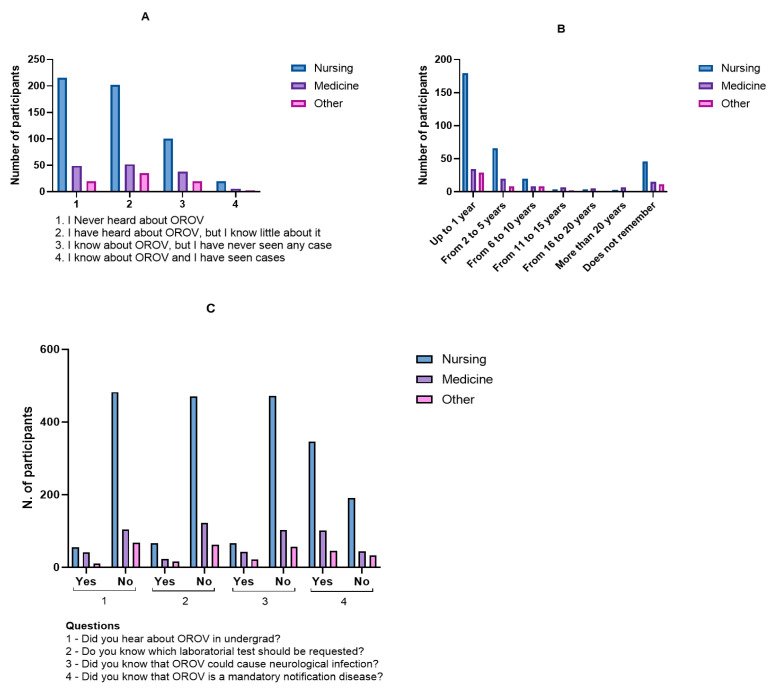
Distribution of participants by years of professional experience and level of knowledge about Oropouche virus. (**A**) Self-reported knowledge about OROV. (**B**) Time since first learning about OROV. (**C**) Knowledge regarding OROV-related training, case notification, diagnostics, and neurological manifestations.

When asked about their level of knowledge of OROV, 284 professionals (37.4%) stated they had never heard of the virus, while 289 (38.0%) had heard of it but knew little. Another 158 (20.8%) reported familiarity with OROV but had never seen clinical cases, and 29 (3.8%) indicated they had seen OROV cases in clinical practice (Figure 2A).

Regarding the time of first contact with information about the virus, 242 participants (31.8%) had learned about it within the past year, 94 (12.4%) between two and five years ago, 36 (4.7%) between six and ten years ago, 13 (1.7%) between eleven and fifteen years ago, 9 (1.2%) between sixteen and twenty years ago, and 10 (1.3%) more than twenty years ago. A total of 72 respondents (9.5%) could not recall when they first learned about OROV (Figure 2B).

A cross-tabulation analysis showed that most participants with limited or no knowledge of OROV had first learned about it within the past year (*n* = 172). A significant proportion of those who were aware of the virus but had no clinical experience also learned about it in the same timeframe (*n* = 59). Overall, the most frequent response was complete unfamiliarity with the virus (*n* = 284; 37.3%).

### 3.3. Educational and Diagnostic Knowledge Gaps

The majority of respondents (*n* = 654; 86.1%) reported that OROV was not covered during their undergraduate education. When asked about compulsory notification, 492 professionals (64.7%) responded correctly, while 268 (35.3%) did not know OROV is a notifiable disease in Brazil.

Regarding diagnostic practices, only 166 participants (21.9%) reported knowing the appropriate laboratory test to request for suspected cases, whereas 594 (78.1%) did not. Concerning neurological complications, 219 professionals (28.8%) were aware of the potential for such manifestations, while the majority (*n* = 541; 71.2%) were not (Figure 2C).

## 4. Discussion

Oropouche fever, a traditionally neglected arboviral disease in Brazil, gained significant attention in 2024 following a marked increase in reported cases and the emergence of serious complications. Reports of fatalities, spontaneous abortions, and microcephaly in newborns have raised concerns within the scientific community and among public health authorities [8,9]. The 2024 epidemiological scenario has exposed vulnerabilities in surveillance systems concerning this virus, underscoring a lack of robust data on its actual incidence, severe clinical manifestations, and potential neurological impacts [10].

Although OROV was first identified in 1955, its circulation in Brazil has long been underestimated, resulting in chronic underreporting. Earlier studies had already warned about the silent spread of the virus, particularly in the Amazon region, where sporadic outbreaks have occurred since the 1960s [1,2]. Nevertheless, Oropouche fever has remained on the margins of discussions about emerging arboviruses, often overshadowed by diseases such as dengue, Zika, and Chikungunya [11].

The recent surge in cases highlights that this is not a novel issue but rather a longstanding public health crisis that has been overlooked. Uncontrolled urbanization, deforestation, and climate change have expanded the habitat of the vector *Culicoides paraensis*, facilitating the spread of OROV into new geographic areas [12]. This complex scenario reinforces the need for a One Health approach that integrates human, animal, and environmental health perspectives [13], especially regarding arboviruses, whose transmission cycles are profoundly influenced by ecological conditions. Poor urban planning, environmental degradation, and climate variability contribute to shifts in epidemiological patterns, increasingly enabling the emergence of previously sylvatic diseases in urban environments [14,15].

Given that no specific antiviral treatment or licensed vaccine is available for Oropouche fever, vector control and personal protective measures remain the cornerstone of prevention [16]. However, early detection and accurate diagnosis play a crucial role in outbreak identification, timely public health response, and differential diagnosis from other febrile illnesses such as dengue, chikungunya, and Zika [17]. The identification of OROV cases, even in the absence of a specific treatment, allows for appropriate case management, vector control strategies, and epidemiological surveillance, which are essential to mitigate transmission.

The results reveal a significant knowledge gap among Brazilian healthcare professionals regarding OROV, including limited clinical recognition and poor understanding of epidemiological and laboratory aspects. This knowledge deficit is reflected in the limited exposure to the virus during undergraduate training, unfamiliarity with its mandatory reporting status, and uncertainty about appropriate diagnostic tests. The observed gaps in epidemiological knowledge among healthcare professionals have important implications for public health. A lack of understanding of OROV’s transmission cycle, vector biology, and geographic distribution can impair timely case identification and outbreak detection, leading to insufficient epidemiological surveillance. This, in turn, increases the risk of sustained virus transmission and hampers effective public health interventions. Therefore, improving epidemiological literacy through targeted training is critical to enhance surveillance accuracy, promote rapid outbreak response, and inform policies aimed at controlling the spread of OROV in Brazil.

This study’s sample, composed of 760 healthcare professionals, predominantly nurses (*n* = 537) and physicians (*n* = 145), enabled a broad assessment of OROV-related knowledge across Brazil. Most responses originated from the Northern region (78%), where the disease is more prevalent, indicating greater exposure to and concern about the virus in this area. Nonetheless, a considerable proportion of professionals in this region reported having never heard of OROV (37.3%), raising concerns about their capacity for early diagnosis and appropriate case management.

Although the majority of participants were female nurses from the northern region of Brazil, this distribution reflects the epidemiological landscape of OROV in the country. The northern region has historically reported the highest incidence of OROV infections and continues to experience recurrent outbreaks. Therefore, while our sample may not fully represent all healthcare professionals in Brazil, it adequately represents the professionals most likely to encounter and manage OROV cases in clinical practice. Nonetheless, we acknowledge that future studies should aim to include broader geographic and professional diversity to improve generalizability.

Our study revealed significant geographic disparities in awareness of Oropouche virus among healthcare professionals in Brazil. Specifically, professionals from southern and southeastern states such as Paraná, Rio Grande do Sul, and São Paulo exhibited the highest proportions of unawareness, with over 60% reporting no prior knowledge of OROV. In contrast, professionals from northern states like Pará and Amazonas, where the virus is endemic, demonstrated higher levels of awareness, with less than 35% unaware of the virus. These findings suggest that regions with historically lower exposure to OROV may require targeted professional training and awareness campaigns to improve preparedness and early detection capacities. Strengthening the knowledge base of healthcare professionals in these areas could enhance diagnostic accuracy, facilitate timely outbreak response, and ultimately reduce the risk of virus spread to new geographic regions.

The fact that 654 participants reported never having heard of OROV during their undergraduate education underscores the urgent need to revise health-related curricula. The literature suggests that emerging arboviruses such as Oropouche fever are often underrepresented in medical and allied health education, undermining epidemiological surveillance and outbreak response [18]. This gap is further evidenced by the 78.2% of respondents who did not know which laboratory test to request for diagnosis, an alarming finding, given that early detection is critical to prevent complications and reduce underreporting.

In 2024, the first fatal cases of acute OROV infection in Brazil and the Americas were described. The deaths occurred in previously healthy young women during an outbreak in southern Bahia. These cases were identified retrospectively, emphasizing the importance of laboratory confirmation for accurate diagnosis, outbreak characterization, and appropriate surveillance [17].

Another relevant finding was the inverse relationship between years of professional experience and knowledge about the virus. Among professionals with fewer than five years since graduation, 49.7% demonstrated no prior knowledge of OROV, indicating insufficient exposure even among recent graduates. This finding underscores the need for continuous education and training, especially as emerging arboviruses become increasingly frequent in Brazil.

A 2013 study assessing nurses’ knowledge of OROV in Pará, a state in the Amazon region, found that only 10% were familiar with the disease, correctly identified its urban distribution, and recognized *Culicoides paraensis* as its main urban vector [unpublished data]. This highlights a long-standing and substantial gap in awareness among healthcare professionals regarding this pathology.

Moreover, the limited perception of OROV’s neurological complications, as reported by 71.2% of respondents, reveals a critical weakness in professional training. Recent literature has identified growing evidence of neurological involvement in OROV infections, including cases of meningitis and encephalitis, which demand increased clinical awareness and proper patient monitoring [19].

The study also revealed a failure in risk communication regarding the notifiable status of the virus. Although OROV is included in Brazil’s list of mandatory notifiable diseases, 35.3% of participants were unaware of this requirement. This gap in knowledge may negatively affect surveillance efforts, hindering the detection of outbreaks and the implementation of timely control measures.

In conclusion, the implementation of effective strategies to enhance OROV-related education among healthcare professionals is urgently needed. This is the first nationwide study in Brazil to formally assess healthcare professionals’ knowledge of Oropouche virus. It reinforces the importance of incorporating this topic into academic curricula, promoting ongoing education campaigns, and strengthening epidemiological surveillance to improve diagnosis, clinical management, and prevention of Oropouche fever in Brazil.

## 5. Conclusions

This study reveals a significant gap in the knowledge and awareness of OROV among Brazilian healthcare professionals. Despite the growing importance of OROV in the Brazilian epidemiological landscape, particularly in light of recent outbreaks and associated complications, healthcare professionals, especially those in endemic regions, exhibit limited understanding of the virus. This lack of knowledge is evident in several critical areas, including clinical recognition, diagnostic procedures, mandatory notification, and the neurological complications associated with the virus.

The study’s findings underscore the urgent need for enhanced training and education about OROV, both during undergraduate education and through ongoing professional development. It is crucial to integrate information about emerging arboviral diseases, such as OROV, into medical and health curricula, as well as to establish targeted education and awareness campaigns for healthcare workers in endemic areas.

Furthermore, strengthening epidemiological surveillance systems and ensuring that all healthcare professionals are aware of the mandatory notification requirements are vital for improving early diagnosis, timely reporting, and effective outbreak control. In the absence of antiviral treatments or vaccines, improved knowledge among healthcare workers is a key element in reducing the burden of Oropouche fever and preventing severe complications, including neurological outcomes.

This study is the first to assess the knowledge of Brazilian healthcare professionals on a national scale, providing valuable insights that can guide public health policy and educational initiatives. Addressing the knowledge gaps identified in this study will contribute to better preparedness for future outbreaks and improve the overall response to arboviral diseases in Brazil.

## Figures and Tables

**Table 1:** Demonstration of absolute numbers and percentage of participants by region and federative state in Brazil.

Region	Federal State	Profession/Gender												Total	%
		Nursing				Medicine				Other					
		Female		Male		Female		Male		Female		Male			
		N.	%	N.	%	N.	%	N.	%	N.	%	N.	%		
North	Amapá	4	0.5	5	0.7	2	0.3	2	0.3	8	1.1	2	0.3	23	3.0
	Amazonas	9	1.2	4	0.5	0	0.0	0	0.0	0	0.0	0	0.0	13	1.7
	Pará	374	49.2	42	5.5	74	9.7	27	3.6	33	4.3	4	0.5	554	72.9
	Rondônia	0	0.0	0	0.0	2	0.3	0	0.0	0	0.0	0	0.0	2	0.3
Northeast	Alagoas	0	0.0	0	0.0	1	0.1	0	0.0	0	0.0	0	0.0	1	0.1
	Bahia	3	0.4	0	0.0	0	0.0	0	0.0	0	0.0	0	0.0	3	0.4
	Ceará	6	0.8	1	0.1	2	0.3	0	0.0	1	0.1	0	0.0	10	1.3
	Maranhão	8	1.1	0	0.0	0	0.0	1	0.1	2	0.3	0	0.0	11	1.4
	Paraíba	2	0.3	0	0.0	2	0.3	0	0.0	0	0.0	0	0.0	4	0.5
	Pernambuco	3	0.4	0	0.0	0	0.0	0	0.0	3	0.4	0	0.0	6	0.8
	Piauí	0	0.0	0	0.0	0	0.0	2	0.3	0	0.0	0	0.0	2	0.3
Midwest	Brasília	3	0.4	1	0.1	2	0.3	0	0.0	4	0.5	0	0.0	10	1.3
	Mato Grosso do Sul	2	0.3	0	0.0	0	0.0	0	0.0	0	0.0	0	0.0	2	0.3
	Mato Grosso	1	0.1	0	0.0	0	0.0	0	0.0	0	0.0	0	0.0	1	0.1
Southeast	Espírito Santo	3	0.4	0	0.0	0	0.0	0	0.0	0	0.0	0	0.0	3	0.4
	Minas Gerais	8	1.1	0	0.0	4	0.5	0	0.0	4	0.5	0	0.0	16	2.1
	Rio de Janeiro	16	2.1	1	0.1	2	0.3	2	0.3	6	0.8	0	0.0	27	3.6
	São Paulo	20	2.6	1	0.1	5	0.7	7	0.9	7	0.9	0	0.0	40	5.3
South	Paraná	2	0.3	0	0.0	0	0.0	0	0.0	3	0.4	0	0.0	5	0.7
	Rio Grande do Sul	10	1.3	0	0.0	1	0.1	6	0.8	0	0.0	0	0.0	17	2.2
	Santa Catarina	8	1.1	0	0.0	1	0.1	0	0.0	1	0.1	0	0.0	10	1.3
**Total**	**21 states**	**482**	**63.4**	**55**	**7.2**	**98**	**12.9**	**47**	**6.2**	**72**	**9.5**	**6**	**0.8**	**760**	**100.0**

## Data Availability

The data presented in this study are available on request from the corresponding author due to ethical reasons.
